# Comparison of COVID-19 Incidence Rates Before and After School Reopening in Israel

**DOI:** 10.1001/jamanetworkopen.2021.7105

**Published:** 2021-04-26

**Authors:** Ido Somekh, Lital Keinan Boker, Tamy Shohat, Massimo Pettoello-Mantovani, Eric A. F. Simões, Eli Somekh

**Affiliations:** 1 Department of Pediatric Hematology Oncology, Schneider Children’s Medical Center of Israel, Petah Tikva, Israel; 2 Sackler Faculty of Medicine, Tel Aviv University, Tel Aviv, Israel; 3 Israel Center for Disease Control, Israel Ministry of Health, Ramat Gan, Israel; 4 University of Haifa School of Public Health, Haifa, Israel; 5 Department of Pediatrics, Scientific Institute Casa Sollievo della Sofferenza, University of Foggia, Foggia, Italy; 6 European Pediatric Association (EPA-UNEPSA), Union of National European, Pediatric Societies and Associations, Berlin, Germany; 7 University of Colorado School of Medicine, Aurora, Colorado; 8 Department of Pediatrics, Mayanei Hayeshuah Medical Center, Bnei Brak, Israel

## Abstract

This cohort study examines COVID-19 incidence rates in youths aged 0 to 19 before and after reopening schools in Israel.

## Introduction

Schools reopened in Israel on September 1, 2020, following summer vacation during active SARS-CoV-2 spread when the incidence of new cases of COVID-19 in Israel was one of the highest in the world. During September 2020, COVID-19 cases further surged in Israel, resulting in school closure (September 14), and a countrywide lockdown. Schools were reopened on November 1. We examined the dynamics in infection rates in children and youths aged 0 to 19 years compared with other age groups, with the goal of understanding whether school reopening was associated with SARS-CoV-2 infection in those aged 0 to 9 years.

## Methods

Because deidentified data from public sources were used, this cohort study was considered exempt from institutional review board approval and informed patient consent was not required. This study followed the Strengthening the Reporting of Observational Studies in Epidemiology (STROBE) reporting guideline.

Daily data were obtained from public national sources of the Ministry of Health,^1,2^ of COVID-19 incidence, SARS-CoV-2 polymerase chain reaction (PCR) tests performed, and the rate of positive samples. SARS-CoV-2 age group–specific weekly incidence rates were calculated and adjusted for the number of tests performed for the following age groups: 0 to 9 years, 10 to 19 years, 20 to 39 years, 40 to 59 years, and 60 years and older. For each age group, incidence rate (weekly number of new cases per 100 000 population of the specific age group) was multiplied by the proportion of this age group in the general population and divided by the proportion of the samples taken from individuals of this age group out of all samples obtained.

Adjusted incidence rate ratios (IRRs) and test positivity rate ratios (RRs) were calculated by comparing mean weekly adjusted incidences and positivity rates in: (1) the first 3 weeks of September, and (2) the weeks during November and December with the last week of August and the last week of October, respectively (weeks that preceded school reopenings) (eAppendix in the Supplement).

We also examined the dynamics of curves of weekly adjusted incidence during the first 3 weeks of September and their slopes. Differences in the incidence, and positivity rates of SARS-CoV-2 PCR tests were analyzed, and *P* values and 95% CIs were determined using 2-proportion *z* tests. *P* < .01 was considered statistically significant. Linear regression was used to generate the slopes statistics. Data analysis was performed using Social Science Statistics software and Excel spreadsheet software version 2019 (Microsoft) from January 2021 to February 2021.

## Results

**Figure 1.**
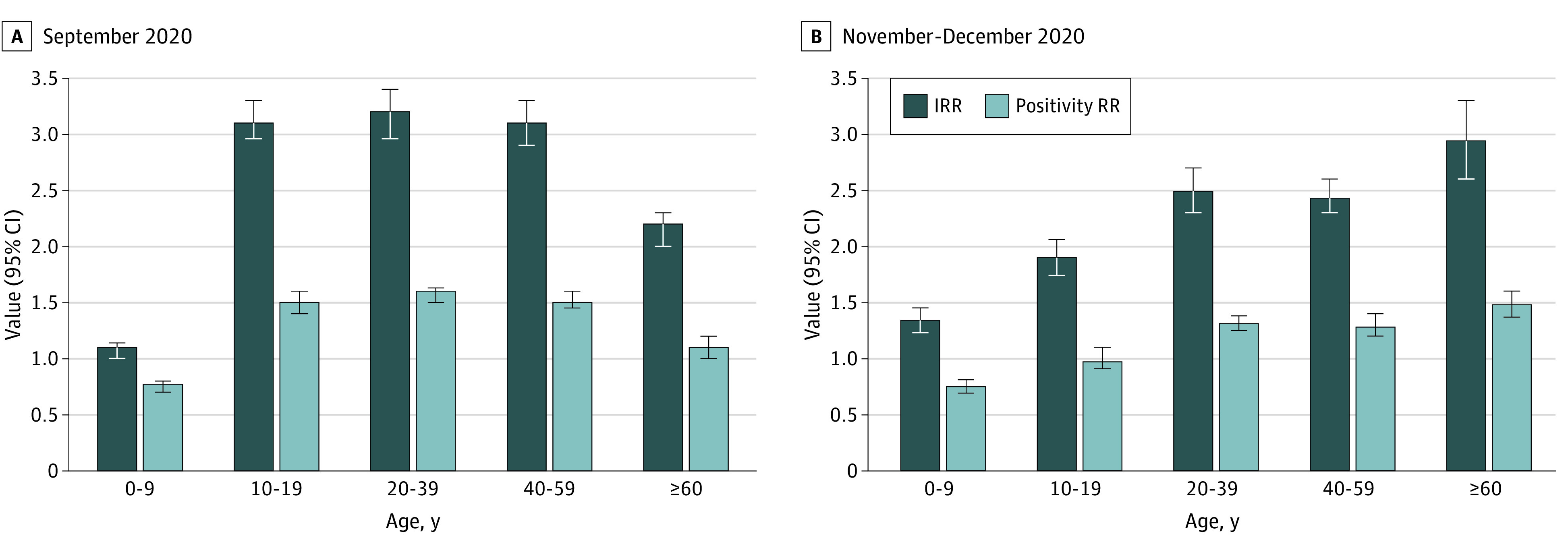
Mean Weekly Increases in Incidence Rate Ratios (IRRs) and Positivity Rate Ratios (RRs) Mean weekly adjusted IRRs and positivity RRs of SARS-CoV-2 tests were calculated by comparing the mean weekly incidence and positivity rates during the following time periods: A, September 1 to September 21, 2020, with those of the week prior to school reopening (August 24-31); and B, November 1 to December 31, 2020, with those of the week prior to school reopening (October 22-29). Error bars indicate 95% CIs.

Data were analyzed from 47 620 children aged 0 to 9 years, 101 304 youths aged 10 to 19 years, 151 295 adults aged 20 to 39 years, 103 056 adults aged 40 to 59 years, and 63 438 adults aged 60 years and older with SARS-CoV-2 infection. Children aged 0 to 9 years had the lowest increase in IRRs (*P* < .001) and in positivity RRs of tests (*P* < .001) during both September (Figure 1A) and November to December (Figure 1B) school attendance periods. For September, the IRRs for the different age groups were 1.1 (95% CI, 1.0-1.14) for those aged 0 to 9 years, 3.1 (95% CI, 2.96-3.3) for those aged 10 to 19 years, 3.2 (95% CI, 2.96-3.4) for those ages 20 to 39 years, 3.1 (95% CI, 2.9-3.3) for those aged 40 to 59 years, and 2.2 (95% CI, 2.0-2.3) for those aged 60 years and older; and the positivity of test RRs were 0.77 (95% CI, 0.7-0.8) for those aged 0 to 9 years, 1.5 (95% CI, 1.4-1.6) for those aged 10 to 19 years, 1.6 (95% CI, 1.5-1.66) for those aged 20 to 39 years, 1.5 (95% CI, 1.45-1.6) for those aged 40 to 59 years, and 1.1 (95% CI, 1.0-1.2) for those aged 60 years and older. For the November to December periods the IRRs for the different age groups were 1.34 (95% CI, 1.23-1.45) for those aged 0 to 9 years, 1.9 (95% CI, 1.74-2.06) for those aged 10 to 19 years, 2.5 (95% CI, 2.3-2.7) for those aged 20 to 39 years, 2.43 (95% CI, 2.3-2.6) for those aged 40 to 59 years, and 2.95 (95% CI, 2.6-3.3) for those aged 60 years and older; and the positivity of test RRs were 0.75 (95% CI, 0.7-0.8) for those aged 0 to 9 years, 0.97 (95% CI, 0.95-1.1) for those aged 10 to 19 years, 1.3 (95% CI, 1.25-1.4) for those aged 20 to 39 years, 1.28 (95% CI, 1.2-1.4) for those aged 40 to 59 years, and 1.48 (95% CI, 1.35-1.6) for those aged 60 years and older.

**Figure 2.**
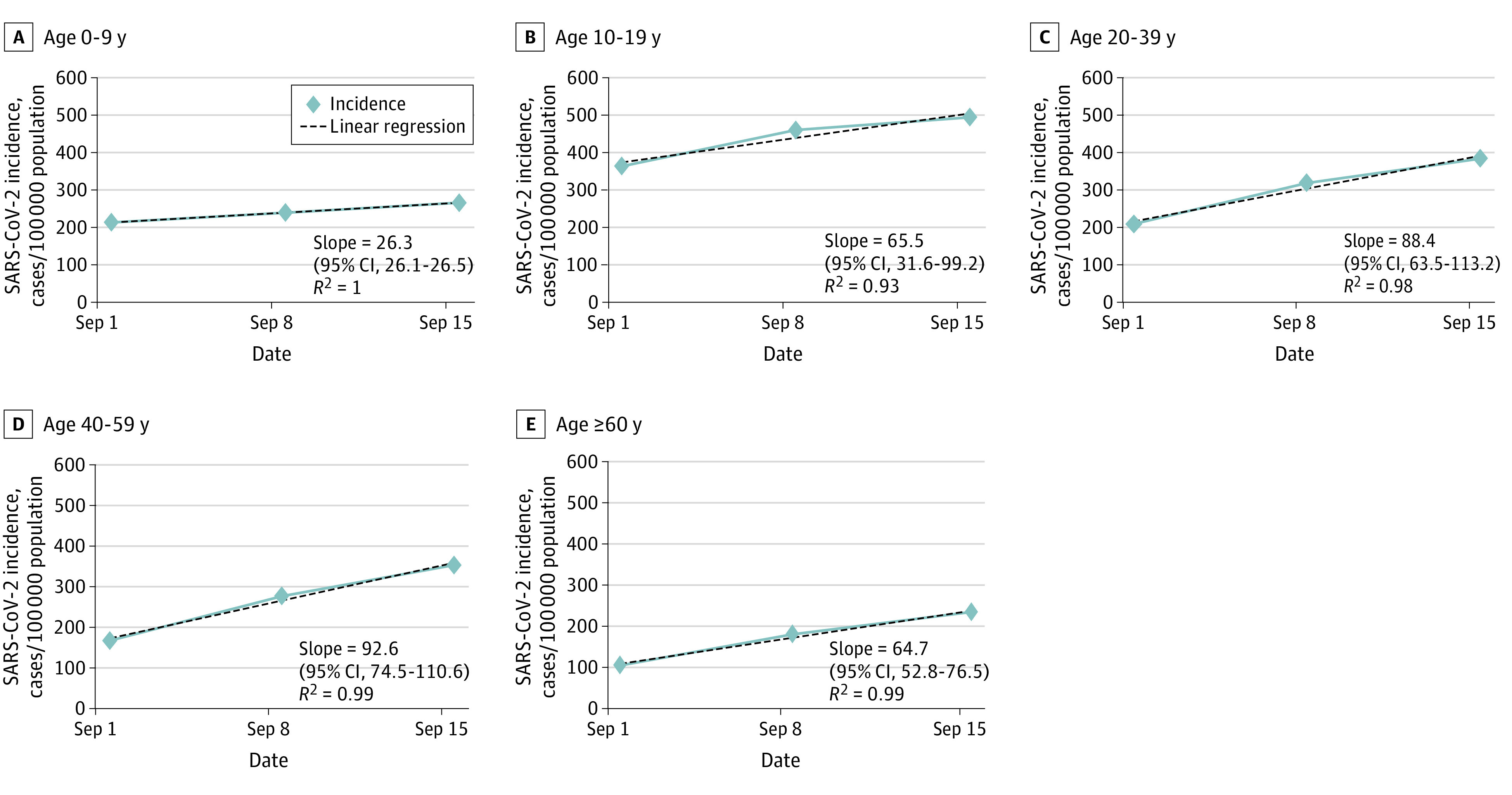
Weekly Adjusted Incidence Curves in Studied Age Groups Weekly adjusted incidence (new SARS-CoV-2 cases per 100 000 population of the specific age group) during September 1 to September 21, 2020. Dates outlined represent day 1 of the studied week. *R*^2^ denotes the *R*^2^ value of the regression line. The slope (with 95% CIs) is for the linear regression line.

Children aged 0 to 9 years had also significantly lower slopes of adjusted incidence of new cases than any other age group during the first 3 weeks of September (Figure 2). The respective slopes were 26.3 (95% CI, 26.1-26.5) for ages 0 to 9 years, 65.5 (95% CI, 31.6-99.2) for ages 10 to 19 years, 88.4 (95% CI, 63.5-113.2) for ages 20 to 39 years, 92.6 (95% CI, 74.5-110.6) for ages 40 to 59 years, and 64.7 (95% CI, 52.8-76.5) for ages 60 years and older.

## Discussion

Children aged 0 to 9 years had the lowest increases in IRRs and in positivity RRs of tests during the 2 school attendance time periods. They also had lower slopes of adjusted incidence curves related to the first 3 weeks of September. These analyses suggest that children in this age group do not have substantial rates of SARS-CoV-2 infection during school attendance and are supported by previous data that demonstrated lower infection rates and lower transmission potential of this age group.^3,4,5,6^

The main limitation of the study is its observational design that cannot inform causal relationships. It is difficult to assess the accurate role of youths aged 10 to 19 because the differences in terms of IRRs and positivity RRs from adults aged 20 to 59 years were insignificantly small. Although it appears that youths aged 10 to 19 years could have actively participated in the spread of infection following school reopening similar to adults aged 20 to 59 years, they could also have been secondary contacts of other sources of infection.

In conclusion, our study’s findings suggest that children aged 0 to 9 years did not have substantial rates of SARS-CoV-2 infection during school attendance periods, and it may be assumed that they did not have a substantial role in COVID-19 spread either during this period. Therefore, resuming school for this age group when lockdown was released appears to have been safe for them. It is probably safer to resume school attendance for youths aged 10 to 19 years only when the epidemic is under control and after implementation of steps to decrease spread in schools.

## Notes

Supplement. **eAppendix.** Supplemental Methods
