# Supplementary material for: Comparison of COVID-19 Incidence Rates Before and After School Reopening in Israel
Source: JAMA Netw Open. 2021 Apr 26;4(4):e217105. doi: 10.1001/jamanetworkopen.2021.7105 (PMC8076960; doi:10.1001/jamanetworkopen.2021.7105)
Supplement: Supplement. — eAppendix. Supplemental Methods [file jamanetwopen-e217105-s001.pdf]

Supplemental Online Content

Somekh I, Boker LK, Shohat T, Pettoello-Mantovani M, Simões EAF, Somekh E. Comparison of COVID-19 incidence rates before and after school reopening in Israel. *JAMA Netw Open.* 2021;4(4):e217105. doi:10.1001/jamanetworkopen.2021.7105

eAppendix. Supplemental Methods

This supplemental material has been provided by the authors to give readers additional information about their work.

© 2021 Somekh I et al. *JAMA Network Open.*

eAppendix. Supplemental Methods:

Age group specific SARS-CoV-2 incidence rate ratios (IRRs) and test positivity rate ratios (RRs) were calculated as follows:

IRR and RR:
SARS-CoV-2 age group specific weekly incidence rates were calculated and adjusted for the number of tests performed for the following age groups: 0-9, 10-19, 20-39, 40-59 and ≥60 years. For each age group, incidence rate (weekly number of new cases /100,000 population of the specific age group) was multiplied by the proportion of this age group in the general population and divided by the proportion of the samples taken from individuals of this age group out of all samples obtained.

IRR and RR for September school reopening:
A: Mean weekly SARS-CoV-2 adjusted incidence (for number of tests performed) in each age group during the first 3 weeks of September.
A1: Mean weekly SARS-CoV-2 positivity rates of samples tested in each age group during the first 3 weeks of September.
a: Weekly SARS-CoV-2 adjusted incidence in each age group during the last week of August (week that preceded school reopening).
a1: Weekly SARS-CoV-2 positivity rates of samples tested in each age group during the last week of August (week that preceded school reopening).
IRR = A/a, RR = A1/a1.

IRR and RR for November -December school reopening:
B: Mean weekly SARS-CoV-2 adjusted incidence (for number of tests performed) in each age group during November -December.
B1: Mean weekly SARS-CoV-2 positivity rates of samples tested in each age group during November -December.
b: Weekly SARS-CoV-2 adjusted incidence in each age group during the last week of October (week that preceded school reopening).
b1: Weekly SARS-CoV-2 positivity rates of samples tested in each age group during the last week of October (week that preceded school reopening).
IRR = B/b, RR = B1/b1.

© 2021 Somekh I et al. *JAMA Network Open.*
